# Effect of Transcutaneous Vagus Nerve Stimulation in Erosive Hand Osteoarthritis: Results from a Pilot Trial

**DOI:** 10.3390/jcm11041087

**Published:** 2022-02-18

**Authors:** Alice Courties, Camille Deprouw, Emmanuel Maheu, Eric Gibert, Jacques-Eric Gottenberg, Julien Champey, Béatrice Banneville, Camille Chesnel, Gérard Amarenco, Alexandra Rousseau, Francis Berenbaum, Jérémie Sellam

**Affiliations:** 1 Service de Rhumatologie, Hôpital Saint-Antoine, Assistance Publique Hôpitaux de Paris (AP-HP), Sorbonne Université, INSERM UMR_S 938, 75012 Paris, France; acourties@yahoo.fr (A.C.); francis.berenbaum@aphp.fr (F.B.); 2 Service de Rhumatologie, Hôpital Saint-Antoine, Assistance Publique Hôpitaux de Paris (AP-HP), 75012 Paris, France; camille.deprouw@aphp.fr (C.D.); emaheu@orange.fr (E.M.); julien.champey@aphp.fr (J.C.); 3 Cabinet de Rhumatologie, 94200 Ivry sur Seine, France; giberteric@wanadoo.fr; 4 Service de Rhumatologie, Centre National de Référence Pour les Maladies Auto-Immunes Systémiques, Hôpital Universitaire de Strasbourg, Université de Strasbourg, 67200 Strasbourg, France; jacques-eric.gottenberg@chru-strasbourg.fr; 5 Service de Rhumatologie, Hôpital Pitié Salpêtrière, Assistance Publique Hôpitaux de Paris (AP-HP), Sorbonne Université, 75013 Paris, France; beatrice.banneville@aphp.fr; 6 GREEN Groupe de Recherche Clinique en Neuro-Urologie, GRC 01, Hôpital Tenon, Assistance Publique Hôpitaux de Paris (AP-HP), Sorbonne Université, 75020 Paris, France; camille.chesnel@aphp.fr (C.C.); gerard.amarenco@aphp.fr (G.A.); 7 Unité de Recherche Clinique de l’Est Parisien (URC-Est), Hôpital Saint-Antoine, Assistance Publique Hôpitaux de Paris (AP-HP), Sorbonne Université, 75012 Paris, France; alexandra.rousseau@aphp.fr

**Keywords:** erosive hand osteoarthritis, pain, function, vagus nerve stimulation, inflammation

## Abstract

Beyond its effect on vegetative functions, the activation of the vagus nerve inhibits inflammation and reduces pain signaling. The aim of this open-label pilot study was to determine the efficacy and tolerance of transcutaneous auricular VNS (taVNS) on erosive hand osteoarthritis (EHOA) symptoms. Symptomatic EHOA patients with hand pain VAS ≥ 40/100 mm and ≥1 interphalangeal swollen joint(s) were included. The taVNS was performed for 4 weeks using an auricular electrode applied one hour per day and connected to a TENS device with pre-established settings. Clinical efficacy was evaluated by changes between baseline and at 4 weeks with hand pain VAS and the functional index FIHOA score, using a Wilcoxon *t*-test. The treatment tolerance was also evaluated. Eighteen patients (median age 69 years old, 83% women) were analyzed. At baseline, hand pain VAS was 60 mm [IQR 50; 78.2] and FIHOA 15 [10.7; 20.2]. After 4 weeks, taVNS significantly reduced hand pain VAS, with a median decrease of 23.5 mm [7.7; 37.2] (*p* = 0.001), as well as FIHOA, with a median decrease of 2 points [0.75; 5.2] (*p* = 0.01). No serious adverse events were reported. One patient stopped taVNS because of auricular discomfort. This first proof-of-concept trial indicated that taVNS is feasible and may decrease joint inflammation and clinical symptoms in EHOA, arguing for a randomized controlled study versus sham stimulation.

## 1. Introduction

Erosive hand osteoarthritis (EHOA) represents around 10% of symptomatic HOA patients, and is more frequently observed in tertiary specialized centers (i.e., 25–55%) [1,2]. EHOA is characterized by joint and systemic inflammation, and is responsible for more severe joint damages and clinical symptoms than non-erosive HOA [3]. However, pharmacological therapeutic options are limited in HOA and recent randomized controlled trials (RCTs) have failed to demonstrate any significant benefits (i.e., TNF inhibitors, hydroxychloroquine or methotrexate, IL-6R blocker) [4,5]. Only one medication (oral prednisolone 10 mg/day) has a greater symptomatic effect than placebo, but raises safety issues [6]. Thus, there is a strong need for therapeutic innovation in EHOA, especially non-pharmacological therapies [7].

The vagus nerve (VN) is the main nerve of the parasympathetic system. In the 2000s, Tracey and colleagues deciphered the “cholinergic anti-inflammatory pathway” in which vagal afferent fibers activate vagal efferent fibers that thereby decrease systemic inflammation. This effect is mediated through the release of acetylcholine binding with one of its receptors, the α-7 nicotinic acetylcholine receptor, expressed on macrophages, and whose activation inhibits cytokine production [8]. Afferent vagal fibers have also been shown to have anti-inflammatory properties in animal arthritis studies, and could have independent analgesic effects since their afferent fibers activate central nervous system centers involved in pain [9,10]. Electrical cervical VN stimulation (VNS) is an approved therapy in refractory epilepsy and depression by the EMA and FDA [11,12]. Interestingly, it has also been investigated as a novel anti-inflammatory strategy. Clinical open-label studies have shown that invasive cervical VNS reduces inflammation and symptoms in rheumatoid arthritis (RA) and Crohn’s disease [13,14]. Invasive VNS has shown promising analgesic effects in fibromyalgia [15]. Traditionally, VNS is performed using an implanted electrical device that is stretched along the cervical branch of the VN and connected to a pulse generator implanted in the chest. This invasive technique is not only expensive, but is also associated with adverse effects, mainly related to the surgical procedure (dysphonia, Horner’s syndrome). Recently, non-invasive techniques of transcutaneous VNS (tVNS) have emerged at different locations of the vagus nerve (i.e., auricular and cervical). Transcutaneous auricular tVNS (taVNS) is performed by applying an electrode to the cymba concha of the left ear [16]. This is done because the ascendant auricular branch of the VN innervates this region [17] and because the left branch has a lesser influence on the sinoatrial node than the right branch [18]. Transcutaneous cymba concha stimulation has been shown to activate the first central relay of the VN (i.e., nucleus tractus solitary) using functional MRI [19,20,21,22]. TaVNS also has confirmed analgesic action in migraine and anti-inflammatory effect in RA patients.

Considering taVNS’s efficacy on pain and inflammation as well as its safety profile, we hypothesized that taVNS using a transcutaneous electrical nerve stimulation (TENS) device could be a new treatment for symptomatic EHOA, a subtype of painful and inflammatory arthritis [23]. This first proof-of-concept feasibility pilot trial aimed to assess the safety and efficacy of a 4-week taVNS treatment on EHOA symptoms.

## 2. Materials and Methods

### 2.1. Study Design and Participants

This was a monocentric open-label study evaluating the efficacy and the safety of taVNS in symptomatic inflammatory EHOA (NCT03919279).

Patients aged ≥18 years, with hand OA defined according to the American College of Rheumatology criteria [24], with ≥1 radiographic erosive (“E” or “R” phases of the Verbruggen–Veys radiographic scoring system) proximal or distal interphalangeal joint (IPJ), and with ≥1 swollen IPJ joint were eligible [25]. These criteria (clinical and radiographic) were checked for each patient by an investigator rheumatology expert in HOA diagnosis and management. Inclusion criteria were hand pain scored on a 0–100 mm visual analog scale (VAS) ≥40/100 mm, ≥1 IPJ symptomatic for more than 3 months, and unresponsive to analgesics or non-steroidal anti-inflammatory drugs (NSAIDs) (or contraindicated).

Exclusion criteria were isolated thumb base OA, other rheumatic diseases, local auricular diseases, ECG abnormalities, symptomatic orthostatic hypotension, or history of recurrent vagal syncope. Patients who were using NSAIDs were told to stop using them at least 48 h before inclusion. The complete list of exclusion criteria is provided in the online Supplementary Table S1.

Since this study is the first one assessing VNS in OA (including EHOA), it was not possible to calculate a sample size. Moreover, due to the pilot design of the trial, we arbitrarily determined the number of patients to be included.

The procedures followed were in accordance with the ethical standards of the responsible committee on human experimentation (Comité de Protection des Personnes OUEST 1 number 2019T2-09) and with the Helsinki Declaration of 1975, as revised in 2000. All patients gave their written informed consent.

### 2.2. Procedures

**Figure 1:**
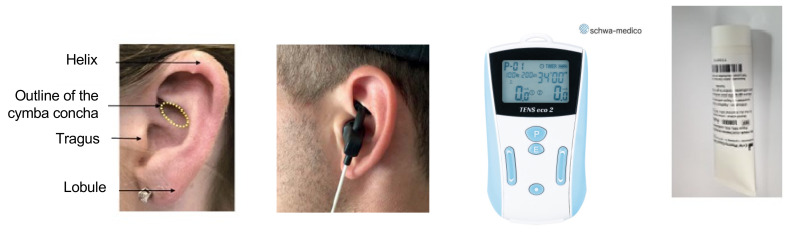
TaVNS device kit. Left to right: Description of the cymba concha, a picture of the auricular electrode applied on the cymba concha, a TENSeco2 device, and the conductive gel (copyright Schwa-Medico).

Before inclusion, patients were given explanations regarding vagus nerve innervation of the ear and the goal of this study. At inclusion, radiographic erosive status was documented by hand radiographs performed within the two years before inclusion and was confirmed by a rheumatologist expert in HOA. ECG and research of orthostatic hypotension were performed before taVNS. TaVNS was performed by the application of an auricular electrode (Schwa Medico, Rouffach, France) placed at the cymba concha of the left ear. The auricular electrode containing the anode was applied with a conductive gel without prior disinfection (bipolar ear electrode from conductive silicone, C+V Pharma Depot GmbH) and was connected to a TENSeco2 device (Schwa Medico, EC certificate DD1379 109-1, software U2:06) (Figure 1). All patients were stimulated 1 h/day, at any time of the day, for 4 weeks. Based on previous studies of fMRI and of taVNS, the TENSeco2 delivered a continuous current, with a biphasic asymmetric balanced waveform, and was set up at 25 Hz frequency and pulse width 50 µs; the intensity was gradually increased to 15 mA or below in case of ear discomfort on the stimulation zone (tingling, dysesthesia) [19,21,26]. The intensity could be adjusted during each daily session of stimulation and had to be set up by the patient every day. After a therapeutic education session at Hospital Saint-Antoine conducted by one of the investigators (AC, CD or JS), the first stimulation was performed at the hospital. Subsequent stimulations were performed at home by the patients themselves with the possibility to contact the research team if questions arose. One follow-up visit was scheduled at 4 weeks. Analgesics other than paracetamol had to be stopped before inclusion and were not permitted during the entire study period. Paracetamol was a rescue medication if necessary and its consumption was quantified throughout the study.

### 2.3. Outcomes

The primary endpoint was 0–100 mm hand pain VAS at 4 weeks. Each patient was asked, ”How much pain in your hand did you experience during the last 48 h?” as recommended by the OARSI [27]. Secondary outcomes were hand function as evaluated by the Functional Index for Hand Osteoarthritis (FIHOA) (0–30) [28,29], the number of painful and swollen joints (trapezo-metacarpal, metacarpophalangeal, proximal and distal IPJ, 0–30), the amount of paracetamol consumed daily, and treatment tolerance at 4 weeks. Patient acceptable symptom state (PASS) was defined by a VAS ≤ 40 mm and the minimum clinically important improvement (MCII) by a decrease of 15 mm out of 100 for absolute improvement, or a 20% pain decrease for relative improvement of their hand pain VAS [30]. For treatment tolerance, patients were asked to report any adverse events (general or local) during the last 4 weeks. Patients could contact the doctor to discuss any adverse events during the study.

### 2.4. Statistical Analysis

Continuous variables are presented as median and interquartile range. Qualitative variables are presented as frequency and percentage. Comparison for hand pain VAS between baseline and 4 weeks was performed using the paired Wilcoxon *t*-test. The comparison also included FIHOA, the number of painful and swollen joints, the cardiac frequency, and blood pressure. Side effects were reported by frequency and percentage. Proportions (95% confidence interval) of patients reaching the PASS and MCII were calculated. All tests were two-sided and a *p*-value < 0.05 indicated statistical significance. Statistical analysis was performed using GraphPad Prism 8.2.1, San Diego, CA, USA.

## 3. Results

Twenty patients were included between May and October 2019. Two patients were lost to follow-up after the first session at hospital (not for safety issues). Thus, 18 patients (83% women) were analyzed.

### 3.1. Efficacy

**Figure 2:**
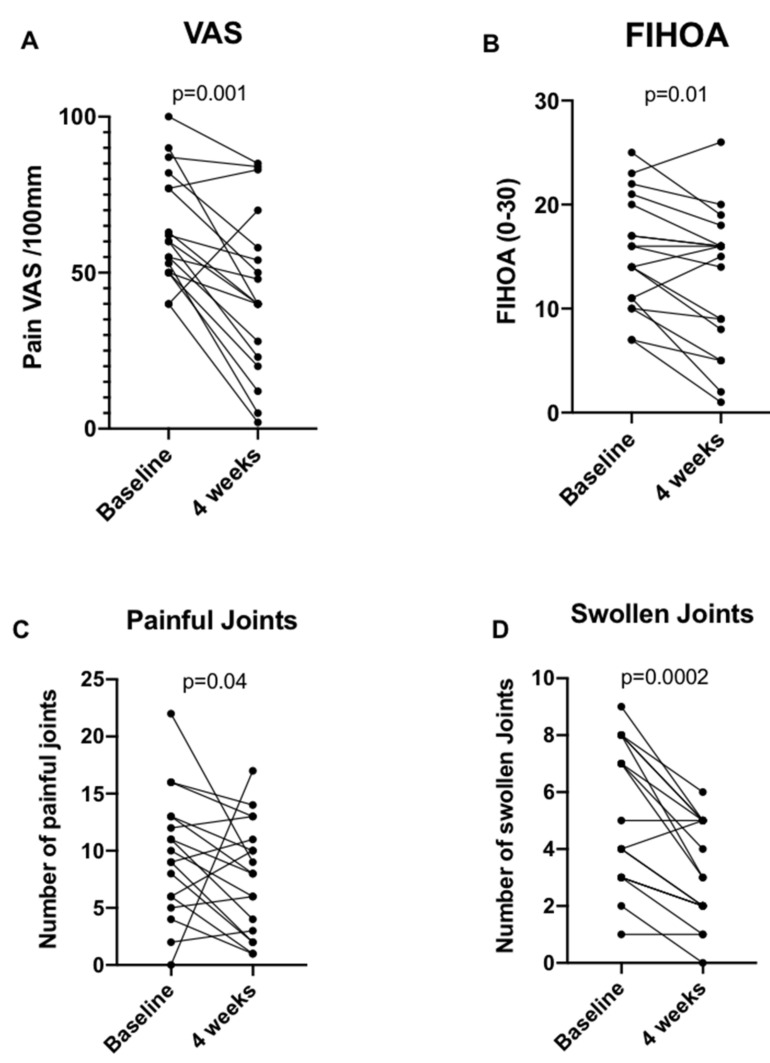
Effect of taVNS on EHOA symptoms. Evolution of (**A**) hand pain VAS on a 0–100 mm scale, (**B**) function evaluated by FIHOA, (**C**) number of painful joints (0–30), (**D**) number of swollen joints (0–30) of erosive hand osteoarthritis for each of the eighteen patients. Paired, non-parametric Wilcoxon *t*-test was used. Abbreviations: VAS, visual analog scale; FIHOA, Functional Index for Hand Osteoarthritis.

Baseline hand pain VAS was 60 mm [50; 78.2] (Table 1). As shown in Table 1 and Figure 2, taVNS significantly reduced VAS pain in 16/18 patients, with a median decrease of 23.5 mm [7.7; 37.2]. Ten of the eighteen patients (55%; CI95%: 0.33–0.75) reached the PASS and thirteen reached the MCII (72%; CI95%: 0.49–0.87). TaVNS also significantly improved the FIHOA score in 14 of the 18 patients with a median decrease of 2 points [0.75; 5.2]. Twelve of the eighteen patients had a reduced number of painful joints with a 3 point [1; 5.2] median decrease, while fifteen patients had a decrease in the number of swollen joints with a median number of 2 joints [1; 3]. Data of each patient are specified in the Supplementary Table S2. Paracetamol consumption was unchanged during the study, with a median baseline consumption of 0.32 g [0.0–5.5], and a median consumption at 1 month of 0 g [0.0–5.5] (*p* = 0.19).

### 3.2. Safety

Eight patients reported 13 adverse events (Table 2). There were no major adverse events throughout the study. Six patients (33%) had local minor symptoms such as tingling (*n* = 3) or local transient pain (*n* = 3). The other adverse events are reported in Table 3. Most of the adverse events were considered minor (*n* = 10) or mild (*n* = 3), and transient (*n* = 8). Three adverse events were not considered related to the device or the stimulation (conjunctivitis, scotoma and floating body left eye). One of the 18 patients stopped taVNS 5 days after the inclusion because of local discomfort. No patient developed orthostatic hypotension.

## 4. Discussion

This is the first proof-of-concept study evaluating the feasibility of VNS in OA. We observed a symptomatic efficacy of taVNS in EHOA. After 4 weeks of stimulation, patients had a significant reduction of pain, painful and swollen joints and of their functional impairment. However, we cannot rule out a placebo effect since there was no control group. Treatment tolerance was good without any serious adverse events, which encourages a randomized and sham-controlled trial.

Since pain and inflammation are typical features of EHOA, we hypothesized that taVNS could be a valuable therapeutic strategy for this OA subtype. After 4 weeks of taVNS, 55% of patients reached the PASS and 72% the MCII for pain, suggesting a symptomatic effect in EHOA. Eight patients reported side effects, but most of them were transient and considered minor. The most frequent adverse event was a local auricular discomfort such as tingling or a local transient pain, which led to only one discontinuation [16]. Such a discomfort might be alleviated by decreasing the time of stimulation in further studies. Three ophthalmological side effects occurred but were considered non-related to taVNS. Although such adverse events have not been reported before, attention should be paid to this type of event in future taVNS trials.

Previous taVNS studies have demonstrated a clinically relevant efficacy in RA, Sjogren syndrome or lupus [31,32,33,34,35,36]. In RA, Addorisio et al. found that vibrotactile taVNS could decrease production of whole-blood LPS-induced cytokines (TNF and IL6) and improve the activity of the disease. They observed a similar range of decrease in the VAS global health of the patient (about 20 mm on a 0–100 mm scale) but they did not report a specific VAS pain score [31]. In systemic lupus, taVNS improved pain over a sham simulation but also relieved fatigue, enhanced patient global assessment, and reduced the number of tender and swollen joints. Our results suggest that taVNS could have both analgesic properties and mitigate inflammatory OA joints, considering the important decrease in the number of swollen joints in 15 of 18 patients at week 4. Concerning safety, we showed in the present study the feasibility of taVNS treatment and satisfying tolerance in this aged population (around 70 years old here). Moreover, two patients discontinued after the first visit since they did not want to participate anymore, and not because of safety issues.

Transcutaneous VNS is a new field of research and can be done at the ear or at the cervical branch [37,38]. Since it has been less evaluated than invasive VNS, some questions remain. Indeed, cadaveric anatomical study has found that ABVN innervates the cymba conchae [17]. Based on fMRI, the cymba concha appears to be, with the inner tragus, the best location to modulate VN at the ear [39]. However, more research is needed to demonstrate a direct ABVN activation.

However, this pilot study has limitations. First, it was an open-label trial and therefore a placebo effect cannot be ruled out—especially with a device such as TENS, which could have the same or a higher placebo effect than oral placebos [40]. Regression to the mean could be also involved in the clinical improvement. These data need to be confirmed in a large randomized trial which is currently ongoing (NCT04520516) with a higher number of participants, a control group (i.e., sham stimulation), and other time points. Furthermore, the anti-inflammatory effect was not assessed biologically by either biomarker use or ultrasound for IPJ synovitis, which are critical to further decipher efficacy in OA [41,42]. This study was a proof-of-concept study to demonstrate the acceptability and feasibility of taVNS in EHOA. Finally, use of a tracker calculating the time of daily use in the device would address the compliance issue related to the device’s use.

## 5. Conclusions

This pioneering proof-of-concept study suggests that 4 weeks of taVNS treatment is feasible, safe and capable of alleviating symptoms and clinical inflammation in subjects with symptomatic EHOA. A randomized controlled trial versus sham stimulation is necessary to confirm these encouraging results for this neglected disease.

## Figures and Tables

**Table 1:** Baseline characteristics of the patients included and analyzed. Abbreviations: FIHOA, Functional Index for Hand Osteoarthritis.

	Baseline Characteristics *n* = 18
Age, years	69 [66.7; 73.2]
Sex Men Women	3 (17%) 15 (83%)
Body mass index, kg/m^2^	22.7 [20.7; 26.3]
Systolic blood pressure, mmHg	129 [116; 139]
Diastolic blood pressure, mmHg	77 [72.7; 79.2]
Cardiac frequency, beats per minute	75 [67.2; 85.5]
Hand Pain VAS, /100 mm	60 [50; 78.2]
Number of painful joints, /30	9.5 [5.7; 13]
Number of swollen joints, /30	4.5 [3; 8]
FIHOA, /30	15 [10.7; 20.2]

**Table 2:** Reported adverse events of 4 weeks of the taVNS in EHOA patients.

Severity	Adverse Event	Number of Patients	Related to Device (Yes/No/Uncertain)	Recovery at 4 Weeks
Minor	Local tingling or pain	6	Yes	Yes for 4/6 patients
	Bilateral conjunctivitis	1	No	Yes
	Scotoma right eye	1	No	No
	Floating body left eye	1	No	Yes
	Auricular device desadaptation of the cymba concha	1	Yes	No
Mild	Insomnia	1	Uncertain	No
	Hand pain when trying to replace the earpiece	1	Yes	Yes
	Post-stimulation fatigue	1	Yes	Yes

**Table 3:** Efficacy of 4 weeks of auricular transcutaneous vagus nerve stimulation on erosive hand OA symptoms. IQR, interquartile range. Paired, non-parametric Wilcoxon *t*-test.

	Baseline *n* = 18	4 Weeks *n* = 18	Median Change Value [IQR] (V1 − V0)	*p*-Value
Systolic blood pressure, mmHg	129 [116; 139]	134 [124; 146.8]	−6 [−17; 5.25]	0.08
Diastolic blood pressure, mmHg	77 [72.7; 79.2]	81 [70; 88.25]	−0.5[−10; 3]	0.09
Cardiac frequency, Beats per minute	75 [67.2; 85.5]	79.5 [69; 87.2]	0.5 [−13; 12.2]	0.82
Hand Pain VAS, /100 mm	60 [50; 78.2]	40 [22.2; 61]	−23.5 [−37.2; 7.7]	0.001
Number of painful joints, /30	9.5 [5.7; 13]	8 [2.7; 11.5]	−3 [−5.2; 1]	0.04
Number of swollen joints, /30	4.5 [3; 8]	3 [2; 5]	−2 [−3; −1]	0.0002
FIHOA, /30	15 [10.7; 20.2]	15.5 [7.2; 16.5]	−2 [−5.2; −0.75]	0.01
Paracetamol consumption (g/week)	0.32 [0.0; 5.5]	0.0 [0.0; 2]	0.0 [−1.25; 0]	0.19

## Data Availability

Data may be obtained upon request to corresponding author.

## Supplementary Materials

The following supporting information can be downloaded at: https://www.mdpi.com/article/10.3390/jcm11041087/s1, Table S1: Exclusion criteria; Table S2: Reported adverse events of 4 weeks of the tVNS in EHOA patients.
