# Supplementary material for: Effect of Transcutaneous Vagus Nerve Stimulation in Erosive Hand Osteoarthritis: Results from a Pilot Trial
Source: J Clin Med. 2022 Feb 18;11(4):1087. doi: 10.3390/jcm11041087 (PMC8878516; doi:10.3390/jcm11041087)
Supplement: Supplementary file 1 [file jcm-11-01087-s001.zip › jcm-1541107-supplementary.pdf]

Supplementary Table S1: Exclusion criteria

Exclusion criteria	• Isolated thumb osteoarthritis<br>• HOA secondary to other known causes (e.g., gout, reactive arthritis, RA, psoriatic arthritis, spondyloarthropathies, septic arthritis)<br>• Skin psoriasis<br>• Current skin disease of the left ear interfering with the application of the auricular electrode for stimulation (eczema, urticarial lesion, skin infection, external otitis, etc.)<br>• Auditory canal not adapted to the application of the ear electrode<br>• Known history of cardiac rhythm disturbances, atrioventricular block > 1st degree, conduction disturbances<br>• Symptomatic orthostatic hypotension or history of recurrent vagal syncope<br>• History of vagotomy<br>• Documented sleep apnea<br>• Existence of a painful syndrome of the upper limbs, which would interfere with assessment of HOA symptoms.<br>• Fibromyalgia<br>• Use of other medical devices electrically active (pacemaker, TENS for chronic pain)<br>• Use of oral, intramuscular or intra-articular or intravenous corticosteroids, immunosuppressants (methotrexate, sulfasalazine, leflunomide, biological disease-modifying antirheumatic drugs), of hyaluronic acid infiltration in the finger joints in the previous 3 months<br>• Any new OA treatment of the hand within the previous month, including physiotherapy and a new digital orthosis.<br>• Hand surgery planned during the study.<br>• Use of any investigational (unapproved) medication within 3 months prior to the screening.<br>• Serious and uncontrolled concomitant disease, including cardiovascular, nervous system, pulmonary, renal, hepatic, endocrine, gastrointestinal or epileptic disease, which, in the opinion of the investigator, would make the study unfeasible.<br>• Pregnant or breastfeeding woman<br>• Patient under legal protection (guardianship or curatorship) and patient deprived of liberty<br>• Participation in other research intervention or in the exclusion period following a previous research if applicable<br>• Patient without social care

	• Taking NSAIDs < 48 hours

Supplementary Table S2: Reported adverse events of 4 weeks of the tVNS in EHOA patients.

Patient number	Adverse event	Related to device (Yes/No/uncertain)	Severity	Recovery at 4 weeks
**2**	Bilateral conjunctivitis	No	Minor	Yes
**6**	Local tingling	Yes	Minor	Yes
**6**	Hand pain when trying to replace the earpiece	Yes	Mild	Yes
**6**	Post-stimulation fatigue	Yes	Mild	Yes
**7**	Local tingling	Yes	Minor	Yes
**7**	Insomnia	Uncertain	Mild	No
**8**	Local tingling	Yes	Minor	Yes
**8**	Auricular device desadaptation of the cymba concha		Minor	No
**12**	Local auricular pain	Yes	Minor	Yes
**13**	Local auricular pain	Yes	Minor	No
**13**	Scotoma right eye	No	Minor	No
**15**	Local auricular pain	Yes	Minor	No
**19**	Floating body left eye	No	Minor	Yes
